# Role of the Glycine Receptor β Subunit in Synaptic Localization and Pathogenicity in Severe Startle Disease

**DOI:** 10.1523/JNEUROSCI.0837-23.2023

**Published:** 2024-01-10

**Authors:** Anna-Lena Wiessler, Ann-Sofie Hasenmüller, Isabell Fuhl, Clémence Mille, Orlando Cortes Campo, Nicola Reinhard, Joachim Schenk, Katrin G. Heinze, Natascha Schaefer, Christian G. Specht, Carmen Villmann

**Affiliations:** ^1^ Institute for Clinical Neurobiology, University Hospital, Julius-Maximilians-University of Würzburg, 97078 Würzburg, Germany; ^2^ Institut National de la Santé et de la Recherche Médicale (Inserm U1195), Université Paris-Saclay, 94276 Le Kremlin-Bicêtre, France; ^3^ Rudolf Virchow Center for Integrative and Translational Bioimaging, Julius-Maximilians-University of Würzburg, 97080 Würzburg, Germany

**Keywords:** glycine receptor, inhibitory synapse, mouse model, oscillator, shaky, startle disease

## Abstract

Startle disease is due to the disruption of recurrent inhibition in the spinal cord. Most common causes are genetic variants in genes (*GLRA1*, *GLRB*) encoding inhibitory glycine receptor (GlyR) subunits. The adult GlyR is a heteropentameric complex composed of α1 and β subunits that localizes at postsynaptic sites and replaces embryonically expressed GlyRα2 homomers. The human GlyR variants of *GLRA1* and *GLRB*, dominant and recessive, have been intensively studied in vitro. However, the role of unaffected GlyRβ, essential for synaptic GlyR localization, in the presence of mutated GlyRα1 in vivo is not fully understood. Here, we used knock-in mice expressing endogenous mEos4b-tagged GlyRβ that were crossed with mouse *Glra1* startle disease mutants. We explored the role of GlyRβ under disease conditions in mice carrying a missense mutation (*shaky*) or resulting from the loss of GlyRα1 (*oscillator*). Interestingly, synaptic targeting of GlyRβ was largely unaffected in both mouse mutants. While synaptic morphology appears unaltered in *shaky* animals, synapses were notably smaller in homozygous *oscillator* animals. Hence, GlyRβ enables transport of functionally impaired GlyRα1 missense variants to synaptic sites in *shaky* animals, which has an impact on the efficacy of possible compensatory mechanisms. The observed enhanced GlyRα2 expression in *oscillator* animals points to a compensation by other GlyRα subunits. However, trafficking of GlyRα2β complexes to synaptic sites remains functionally insufficient, and homozygous *oscillator* mice still die at 3 weeks after birth. Thus, both functional and structural deficits can affect glycinergic neurotransmission in severe startle disease, eliciting different compensatory mechanisms in vivo.

## Significance Statement

Glycinergic dysfunction results in a neurological disorder called startle disease. The most common affected gene is *GLRA1* that encodes the glycine receptor α1 subunit. A comprehensive analysis of the role of GlyRβ in startle disease has been hampered by the lack of reliable GlyRβ-specific antibodies. The novel mouse model *Glrb*^eos^ has allowed us to identify compensatory processes during pathology. Targeting of heteromeric GlyRαβ receptors to synaptic sites is maintained in the presence of mutated GlyRα1 and even in the absence of GlyRα1. Thus, insufficient quality control of mutant heteromeric GlyRs in the endoplasmic reticulum (ER) underlies the ineffectiveness of functional compensation, even though the presence of a mature postsynaptic specialization could serve as a structural platform to overcome disease severity.

## Introduction

Hyperekplexia (OMIM147100, startle disease) is a rare neurological motor disorder (Becker, 1995). The symptoms are severe and sometimes life-threathening including massive rigidity, high muscle tonus, and excessive startle to unexpected acoustic or tactile stimuli. The disease is caused by mutations in genes encoding glycine receptor (GlyR) subunits or the glycine transporter 2 (GlyT2). GlyRs enable fast inhibitory neurotransmission in the adult brainstem (BS) and spinal cord (SC) and are important for proper motor control (Lynch, 2004). They belong to the superfamily of cys-loop receptors and are pentameric ligand-gated chloride channels. Each subunit consists of a large N-terminal extracellular domain (ECD), four transmembrane (TM) domains, and a short extracellular C-terminus (Du et al., 2015; Huang et al., 2015). Four α (α1–α4) and one β subunit of GlyRs have been identified. GlyRα subunits can form homomeric pentamers located at presynapses or extrasynaptic sites (Turecek and Trussell, 2001; Xiong et al., 2014), whereas GlyRβ can only form functional ion channels when co-assembled with GlyRα in various subunit stoichiometries (Durisic et al., 2012; Patrizio et al., 2017; Yu et al., 2021; Zhu and Gouaux, 2021). The β subunit directs GlyRs to synapses due to a direct interaction with the scaffold protein gephyrin (Kneussel and Betz, 2000). The GlyRβ subunit has been associated with anxiety and panic disorders (Deckert et al., 2017; Schaefer et al., 2020). GlyRβ mRNA was found to be widely expressed throughout the SC and the brain (Malosio et al., 1991). A non-commercial monoclonal antibody has been generated, enabling the detection of GlyRβ at the protein level in SC, BS, and midbrain (Weltzien et al., 2012).

During development, homomeric GlyRα2 represents the major form of GlyRs. A subunit switch after birth has been described resulting in the expression of mainly GlyRα1β complexes in adult BS nuclei and SC. In contrast, α2 expression decreases after a peak at postnatal days 7–10 (Liu and Wong-Riley, 2013; Morelli et al., 2017). Mutations in the genes *GLRA1* (GlyRα1), *GLRB* (GlyRβ), and *SLC6A5* encoding GlyT2 are the major underlying causes of startle disease in humans and rodents (Rees et al., 2006; Chung et al., 2010, 2013; James et al., 2013; Bode and Lynch, 2014; Schaefer et al., 2015; Piro et al., 2021). Most mutations were found in *GLRA1* for which different mouse models exist. The mouse model *oscillator* represents a GlyRα1 structural null mutation. A microdeletion leads to an early STOP codon resulting in a complete loss of GlyRα1 protein expression. Homozygous *oscillator* mice show severe symptoms starting around P14 and die at 3 weeks of age (Buckwalter et al., 1994; Kling et al., 1997). Whether the lack of GlyRα1 has an influence on the GlyRβ expression and/or receptor localization remains unclear. Interestingly, in heterozygous *oscillator* mice, the packing density of GlyRs at SC synapses with a density of ∼2,000 receptors/µm^2^ did not change compared to wild-type animals. Yet, significant alterations were observed regarding the size and morphology of glycinergic synapses (Maynard et al., 2021). The effect of the more severe, homozygous *oscillator* mutations on glycinergic synapses has not yet been analyzed. *Shaky*, another severe startle disease model, carries the missense mutation Q177K in the β8–β9 loop of the GlyR ECD. Symptoms in homozygous *shaky* mice start at P16 and increase during the following week, but the mice die somewhat later, at ∼4–6 weeks. It has been suggested that *shaky* underlies altered GlyRα1 synaptic targeting (Schaefer et al., 2017); however, the role of the GlyRβ subunit in this context is not yet understood.

In this study, we make use of a newly generated knock-in *Glrb*^eos^ mouse line that allows the specific detection of GlyRβ via the mEos4b fluorescence tag (Maynard et al., 2021). This reporter mouse was crossed with two different startle disease lines, namely *oscillator* and *shaky*, to investigate the role of GlyRβ in the pathogenicity as a result of functional and/or structural GlyRα1 deficiency.

## Materials and Methods

### PCR genotyping

Genotyping of the *Glrb*^eos^ mouse line (*Glrb*^tm1(Eos4)Ics^, MGI:6331065) and two hybrid mouse lines *Glra1*^spdot^/*Glrb*^eos^ (*oscillator*, JAX stock #000536, RRID:IMSR_JAX:000536) and *Glra1*^shy^/*Glrb*^eos^ [*shaky*, (Schaefer et al., 2017); C. Paige, University Health Network Research, Toronto, Canada] was performed as described in Maynard et al. (2021) and Schaefer et al. (2018), respectively.

### Immunohistochemical stainings of brain and spinal cord tissue

Brains and SCs were extracted from deeply anesthetized *Glra1*^+/+^/*Glrb*^eos/eos^ and *Glra1*^spdot/spdot^/*Glrb*^eos/eos^ mice, embedded in Tissue-Tek (4583, Sakura Finetek) and immediately frozen on dry ice. A cryostat (CM1950, Leica) with a chamber temperature of −20°C was used to cut sections of 9 µm thickness. The sections were mounted on SuperFrost Plus slides (03-0060 Langenbrinck) and directly used for stainings or stored at −80°C. The sections were fixed with ice-cold 2% paraformaldehyde (PFA) in phosphate-buffered saline (PBS) pH 7.4 for 30 s at room temperature (RT). After washing in PBS pH 7.4, the sections were shortly dipped in 50 mM NH_4_Cl for quenching and incubated in 0.1 mM glycine for 30 min. For blocking, a 10% normal goat serum (NGS) in PBS pH 7.4 was used followed by a primary antibody incubation with anti-mEos-Cy3 (N3102-SC3-L, 1:200, NanoTag), GlyRα1 (146111, RRID:AB_887723, 1:500, Synaptic Systems), GlyRα2 (NBP3-03685, 1:1,000, Novus Biologicals), and gephyrin (147111, RRID:AB_887719, 1:500, Synaptic Systems) in blocking solution over night (ON) at 4°C. The secondary antibody goat-anti-mouse-Cy5 (115-175-146, Dianova) diluted 1:500 in blocking solution was incubated for 1 h in the dark at RT, and the nuclei were stained with 4’,6-diamidino-2-phenylindole (DAPI) in PBS pH 7.4 for another 10 min. The sections were mounted in FluorSave reagent (345789, Calbiochem) and sealed with a glass coverslip.

### Spinal cord tissue preparation for spinal cord clearing and photoactivated localization microscopy

Mice were perfused with 4% w/v PFA (Polysciences, EM grade) and 0.1% v/v glutaraldehyde (GA; CliniSciences) in PBS pH 7.4. Perfused animals were kept on ice for 30 min, followed by the dissection of the brain and SC in PBS. Tissue was post-fixed in 4% w/v PFA in PBS ON at 4°C, rinsed in PBS, and kept in PBS containing 30% w/v sucrose ON at 4°C.

The SCs were cut into smaller segments of thoracic and lumbar regions and either used for the SC clearing protocol or embedded in Neg-50 medium (6502G, Thermo Scientific) before being frozen and stored at −80°C. A cryostat (CM3050S, Leica) with a chamber temperature of −23°C was used to cut sections of 4 µm nominal thickness. The sections were mounted in PBS on SuperFrost Plus slides (J1800AMNZ, Thermo Scientific), immunolabeled, covered with a glass coverslip (type #1.5, 0.17 µm thickness, catalog #6310134, VWR), and sealed with silicone polymer (picodent twinsil speed 22). Alternatively, slices were cut from freshly-frozen SC in Tissue-Tek, placed on glass coverslips, post-fixed with 2% PFA, labeled, and directly used for photoactivated localization microscopy (PALM) imaging in an open chamber in PBS.

### Spinal cord clearing

Spinal cord tissue-clearing was performed following the recently published ultimate DISCO (uDISCO) method (Pan et al., 2016) as it combines good clearing capability with the preservation of mEos4b fluorescent signals.

### Primary spinal cord neurons

Mixed SC neurons were prepared from the different mouse lines at the embryonic day 12–13. The experiments were approved by the local veterinary authority (Veterinäramt der Stadt Würzburg) and the Ethics Committee of Animal Experiments, that is, Regierung von Unterfranken (license no.:55.2.2-2532.2-949-31). Neuronal cultures were prepared as decribed previously (Fischhaber et al., 2021). Genotyping was performed for each embryo individually as described above. Staining was done after 16–18 days in vitro (DIV16-18).

### Immunocytochemical stainings

#### Stainings of spinal cord neurons

Cells and cultured neurons on glass coverslips were fixed with 4% PFA/4% sucrose in PBS pH 7.4 for 20 min at RT. Afterwards, the cells were blocked and permeabilized with 5% NGS/0.2% Triton X-100 in PBS for 30 min. The primary antibodies against GFP (SC8224, RRID:AB_2276004, 1:500, Santa Cruz), gephyrin (147011, RRID:AB_887719, 1:500, Synaptic Systems), GlyRα1 (146111 or 146118, RRID:AB_887723 or RRID:AB_2832240, 1:500, Synaptic Systems), pan-α-GlyR (146011, RRID:AB_887721, 1:250, Synaptic Systems), and anti-mEos-488 (N3102-At488, 1:200, NanoTag) in blocking solution were incubated for 1 h, followed by incubation with the secondary antibodies goat-anti-rabbit-Alexa-488 (111-546-003, RRID:AB_2338053, Dianova), goat-anti-mouse-Cy3 (115-165-003, RRID:AB_2338680, Dianova), goat-anti-rabbit-Cy3 (111-165-003, RRID:AB_2338000, Dianova), goat-anti-mouse-Cy5 (115-175-146, Dianova), or goat-anti-rabbit-Cy5 (111-175-006, Dianova) diluted 1:500 in a blocking solution for 1 h in the dark. The cell nuclei were stained with DAPI in PBS for 5 min, and the cells were mounted on glass slides in Mowiol.

#### Immunostaining of spinal cord tissue slices used for PALM

Fixed SC slices were permeabilized and blocked in PBS containing 4% w/v BSA (A7030, Sigma) and 0.25% v/v Triton X-100 for 1 h at RT. A primary anti-gephyrin antibody (mAb7a, 147011, RRID:AB_887719, Synaptic Systems) was applied at a dilution of 1:500 in blocking solution (PBS with 1% BSA and 0.06% Triton X-100) for 2.5 h at RT. After three washes for 5 min in PBS, the secondary antibody goat-anti-mouse-IgG-Alexa-647 (A21236, Invitrogen) was applied at a 1:1,000 dilution in blocking solution for 90 min in the dark at RT. The sections were mounted in PBS and sealed with picodent twinsil or directly imaged in an open chamber in PBS. The following are the acquisition parameters of conventional fluorescence images (Zeiss ELYRA PS.1 setup): 10 image frames of 50 ms exposure were taken at 488 nm with 0.2% laser intensity (nominal laser power: 300 mW) and a camera gain of 300 to record the unconverted (green) mEos4b-GlyRβ fluorescence. Gephyrin immunofluorescence was detected in the far red channel, by taking 10 images of 100 ms with the 642 nm laser (nominal laser power: 150 mW) at 5% output and a camera gain of 200. The stacks of images were averaged, resulting in a single image for each fluorescence channel.

### Western blot

The SC, BS, and cortex (CX) of deeply anaesthesized 2- to 4-week-old *Glra1*^+/+^/*Glrb*^eos/eos^ and *Glra1*^spdot/spdot^/*Glrb*^eos/eos^ animals of both sexes were extracted and frozen at −80°C. Lysates were prepared using a glass-homogenizer in 1 ml homogenization buffer [in mM: 20 HEPES, 100 potassium acetate, 40 KCl, 5 EGTA, 5 MgCl_2_, 5 DTT, 1% Triton X-100, 1 PMSF (freshly added), protease inhibitors (Roche)]. After a 15 min centrifugation at 10,000 × *g* at 4°C, the supernatant containing the protein samples was transferred, and 20 µg of protein was used for Western blotting.

For SDS-PAGE, 11% polyacrylamide gels were freshly prepared, followed by Western blot on nitrocellulose membranes (GE Healthcare). The membranes were blocked for 1 h with 5% BSA in TBS-T (TBS with 1% Tween 20). The primary antibodies against different glycine receptor subunits were GlyRα1 (AB5052, RRID:AB_91659, 1:1,000, Merck), GlyRα2 (NBP3-03685, 1:1,000, Novus Biologicals), and pan-α-GlyR (146008, 1:1,000, Synaptic Systems). The anti-GAPDH (CB1001, 1:1,000, Merck) served as a loading control. Following a 1 h incubation with a horseradish peroxidase-coupled secondary antibody (Dianova, 111-036-003, RRID:AB_2337942 and 115-035-146, RRID:AB_2307392, 1:15,000) at RT, immunoreactivity was detected through chemiluminescence using Clarity Western ECL substrate (Clarity Western Peroxide Reagent, BioRad 170-5061). Data analysis was performed using Fiji software (Schindelin et al., 2012).

### Electrophysiological recordings

Patch-clamp whole-cell recordings at RT were performed using recording pipettes pulled from borosilicate capillaries with open resistances of 3.5–5.5 MΩ and filled with internal buffer (in mM: 140 CsCl, 1 EGTA, 10 HEPES, 6 D-glucose; pH 7.2, adjusted with CsOH). Current amplitudes and dose–response curves (EC_50_ values) were measured by the application of glycine in a concentration series of 10–600 µM in the external buffer (in mM: 130 NaCl, 3 KCl, 1.5 CaCl_2_, 2 MgCl_2_, 10 HEPES, 6 D-glucose, 10 TEA-Cl; pH 7.35, adjusted with NaOH). Glycine solutions were applied by the OctaFlow II system (ALA Scientific Instruments). To ensure that heteromeric GlyRs were expressed by the neurons, 100 µM glycine was applied in the external buffer supplemented with 50 µM picrotoxinin (Sigma Aldrich) at the end of the recordings. When current amplitudes were blocked with picrotoxinin by more than 50% compared to 100 µM glycine application alone, cells were excluded from analysis since homomeric GlyRs show a higher sensitivity to picrotoxinin (Pribilla et al., 1992). Current responses were amplified with an EPC-10 amplifier (HEKA Elektronik) and measured at a holding potential of −60 mV using Patchmaster Next software (HEKA Elektronik).

### Fluorescence imaging, image processing, and analysis

The images of immunocytochemical stainings in cultures were captured using an confocal Olympus FluoView ix1000 microscope (Olympus) with an UPLSAPO 60× oil objective (N.A. 1.35), the software FluoView FV 1000, and diode lasers of 405 nm, 495 nm, 560 nm, and 647 nm wavelengths. The immunohistochemical stainings in tissue were captured by a wide-field Zeiss Axio Imager 2 microscope (Zeiss) with a 20× air objective (N.A. 0.8), the ZEN software 2.6, and light sources of 385 nm, 469 nm, 555 nm, and 630 nm wavelengths. All images were captured in 1,024 × 1,024 pixels and 16 bit.

We used the Fiji software for image processing and analysis. Puncta per 100 µm dendrite length were analyzed via the plugins NeuronJ and SynapcountJ. With NeuronJ, dendrites were traced in the gephyrin channel. Puncta were counted with SynapcountJ for every individual channel and all combinations of colocalization of two channels by using the dendrite mask from NeuronJ and a diameter of 10 pixels for dendrite thickness. The thresholds were 132 for single channels and 60–120 for GlyRβ–gephyrin, 60–132 for GlyRβ–GlyRα, and 60–132 for GlyRα–gephyrin. The extrasynaptic puncta were calculated by subtracting the puncta that colocalize with gephyrin from the whole amount of protein puncta.

### 3D-microscopy and image processing

#### Photoconversion and confocal image acquisition

Photoconversion was performed prior to imaging with a laser scanning confocal microscope (TCS-SP8 Leica), operated by LAS X (3.1.5.16308) software, equipped with a HC PL0-95 FLUOTAR 10×/0.30 DRY objective using a 405 nm laser at 0.2% intensity. The photoconversion was performed at 400 Hz, zoom 0.95, in a region of 1.22 × 1.22 × 0.23 mm (voxel dimensions 2.40 × 2.40 × 0.13 µm).

Subsequently, imaging was performed by exciting mEos4b-GlyRβ at 561 nm and detecting the fluorescence emission by a Hybrid detector in the range from 570 to 630 nm. Images were collected at 400 Hz, zoom 0.95, region of 1.22 × 1.22 × 0.83 mm (voxel dimensions 0.38 × 0.38 × 3.00 µm).

#### Image processing

The raw image was deconvolved using the Deconvolution Wizard from Huygens Professional 22.10 (Scientific Volume Imaging). Briefly, the SNR was automatically set to 1.5, and the estimated background was automatically set to 0 using a radius of 1 µm. Using an acuity of 150, 16 iterations were needed to meet the quality criterion of 0.01.

Next, the image was segmented using Imaris 10.0.0 (Bitplane AG) to remove objects outside the main SC. The surface tool was applied with a smoothing radius of 10 µm and objects smaller than 1.1 × 10^7^ voxel were removed.

### Photoactivated localization microscopy

Single molecule-based super-resolution imaging (PALM) was carried with an ELYRA PS.1 (Zeiss) microscope setup. Samples were placed on the motorized XY scanning stage of the Axio Observer.Z1 SR inverted microscope, and imaged with a Plan-Apochromat 100× oil-immersion objective (N.A. 1.46) with an additional 1.6× lens in the emission path, using an Andor iXon 897 back-thinned EMCCD camera (16 bit, 512 × 512 pixels, 16 µm pixel size, QE 90%), resulting in a final image pixel size of 100 nm. For wide-field imaging in the green channel, we used a 488 nm excitation laser (300 mW) and a BP 495-575 + LP 750 emission filter. PALM imaging in the red channel was done with a 405 nm laser (50 mW) for photoconversion and a 561 nm laser (200 mW) for excitation; the emitted light was filtered with a BP 570-650 + LP 750 filter. Images were acquired with Zen software (Zeiss, Zen 2012 SP5 FP3 black, 64 bit).

PALM acquisition parameters: First we identified glycinergic synapses in the gray matter of the SC slices with the 488 nm laser at low intensity. Reference images of the unconverted (green) mEos4b-GlyRβ fluorescence were taken with an exposure time of 100 ms (≥10 frames, image size 256 × 256 pixels) and averaged. PALM movies of 20,000 frames were then recorded with constant 561 nm laser illumination (output power 80%, 50 ms streamed acquisition, EMCCD gain 300). Photoconversion of mEos4b-GlyRβ was done by continuous 405 nm laser illumination (gradually increased from 0.01 to 5% intensity by frame 20,000).

### PALM image analysis

The PALM movies were analyzed with Zen software using the following parameters: a mask size of 9 pixels for peak detection, an intensity threshold of 6 (signal to noise ratio), and with exclusion of overlapping molecules. The images were drift corrected by temporal correlation of the detection coordinates, and rendered super-resolution images were generated by representing each detection with a two-dimensional Gaussian distribution with a width *σ* corresponding to 0.8× the localization precision of each detection and a pixel size of 10 nm. We used the spot detector plugin from Icy software (https://icy.bioimageanalysis.org, v. 2.4.3.0, Institut Pasteur, parameters: scale 3 and scale 4, sensitivity 100, size filtering 100–2,000 px) to detect dense clusters of mEos4b-GlyRβ in the rendered images, likely representing glycinergic synapses. The output data consist in a table with the mean size of the synaptic clusters and the number of ROIs detected in each image.

### Experimental design and statistical analysis

All experiments were performed with at least three different male or female animals per genotype. The numbers of experiments/animals and dendrites for synaptic density analysis are displayed in Tables 1–3. We used Graph Pad Prism for statistical analysis. Data are represented as mean ± S.E.M (standard error of the mean). Outliers were identified by ROUT (*Q* = 1%) and removed. Normality of the data was reviewed by Shapiro–Wilk normality test (*α *= 0.05). Statistical significance was calculated using an unpaired two-tailed Mann–Whitney test, a Kruskal–Wallis test, a one-way *ANOVA* with Dunnetts multiple comparison test, or an unpaired *t* test depending on the data set. All *p*-values are given in the Results part and Tables 1–3. The 0-hypothesis was rejected at a level of *p* < 0.05.

**Table 1.** Quantitative analysis of synaptic density of Glra1^+/+^ and Glra1^spdot/spdot^ neurons

Protein colocalization	Genotype	Synaptic density per 100 microns	Significance and *p*-values	*n* = number of dendrites	*N* = number of experiments
GlyRβ	*Glra1*^+/+^/*Glrb*^eos/eos^	42.92 ± 1.89	*p = 0.0275	102	3
	*Glra1*^spdot/spdot^/*Glrb*^eos/eos^	37.44 ± 1.99		99	3
GlyRα	*Glra1*^+/+^/*Glrb*^eos/eos^	35.21 ± 1.64	**p = 0.0060	109	3
	*Glra1*^spdot/spdot^/*Glrb*^eos/eos^	45.73 ± 2.46		99	3
Gephyrin	*Glra1*^+/+^/*Glrb*^eos/eos^	40.57 ± 2.02	n.s. *p *= 0.4663	109	3
	*Glra1*^spdot/spdot^/*Glrb*^eos/eos^	37.44 ± 1.70		100	3
GlyRβ–GlyRα	*Glra1*^+/+^/*Glrb*^eos/eos^	30.13 ± 1.25	**p = 0.0072	102	3
	*Glra1*^spdot/spdot^/*Glrb*^eos/eos^	38.58 ± 2.16		95	3
GlyRβ–gephyrin	*Glra1*^+/+^/*Glrb*^eos/eos^	39.52 ± 1.90	n.s. *p *= 0.1976	109	3
	*Glra1*^spdot/spdot^/*Glrb*^eos/eos^	36.33 ± 1.94		100	3
GlyRα–gephyrin	*Glra1*^+/+^/*Glrb*^eos/eos^	32.04 ± 1.36	n.s. *p *= 0.7803	99	3
	*Glra1*^spdot/spdot^/*Glrb*^eos/eos^	32.69 ± 1.51		96	3
Extrasynaptic GlyRβ	*Glra1*^+/+^/*Glrb*^eos/eos^	16.04 ± 1.47	n.s. *p *= 0.5913	61	3
	*Glra1*^spdot/spdot^/*Glrb*^eos/eos^	17.48 ± 1.72		51	3
Extrasynaptic GlyRα	*Glra1*^+/+^/*Glrb*^eos/eos^	14.59 ± 1.21	***p = 0.0009	71	3
	*Glra1*^spdot/spdot^/*Glrb*^eos/eos^	25.34 ± 2.30		65	3

Significance values: *p < 0.05; **p < 0.01, ***p < 0.001; n.s. = not significant.

**Table 2.** Quantitative analysis of western blots signals of Glra1^+/+^ and Glra1^spdot/spdot^ mice

Protein	Genotype	Tissue	Expression level	Significance and *p*-values	*N* = number of experiments
GlyRα1	*Glra1*^+/+^/*Glrb*^eos/eos^	SC	0.80 ± 0.05	****p < 0.0001	3
	*Glra1*^spdot/spdot^/*Glrb*^eos/eos^		0.002 ± 0.001		3
	*Glra1*^+/+^/*Glrb*^eos/eos^	BS	0.79 ± 0.04	****p < 0.0001	3
	*Glra1*^spdot/spdot^/*Glrb*^eos/eos^		0.006 ± 0.004		3
	*Glra1*^+/+^/*Glrb*^eos/eos^	CX	0.001 ± 0.001	n.s. *p *= 0.0688	3
	*Glra1*^spdot/spdot^/*Glrb*^eos/eos^		0.001 ± 0.001		3
GlyRα	*Glra1*^+/+^/*Glrb*^eos/eos^	SC	1.37 ± 0.13	**p = 0.0042	3
	*Glra1*^spdot/spdot^/*Glrb*^eos/eos^		3.88 ± 0.41		3
	*Glra1*^+/+^/*Glrb*^eos/eos^	BS	1.58 ± 0.44	n.s. *p *= 0.0646	3
	*Glra1*^spdot/spdot^/*Glrb*^eos/eos^		3.68 ± 0.70		3
	*Glra1*^+/+^/*Glrb*^eos/eos^	CX	0.21 ± 0.06	n.s. *p *= 0.0957	3
	*Glra1*^spdot/spdot^/*Glrb*^eos/eos^		0.38 ± 0.06		3
GlyRα2	*Glra1*^+/+^/*Glrb*^eos/eos^	SC	0.77 ± 0.14	*p = 0.0166	3
	*Glra1*^spdot/spdot^/*Glrb*^eos/eos^		1.64 ± 0.17		3
	*Glra1*^+/+^/*Glrb*^eos/eos^	BS	0.83 ± 0.07	*p = 0.0157	3
	*Glra1*^spdot/spdot^/*Glrb*^eos/eos^		1.37 ± 0.12		3
	*Glra1*^+/+^/*Glrb*^eos/eos^	CX	0.35 ± 0.02	n.s. *p *= 0.0569	3
	*Glra1*^spdot/spdot^/*Glrb*^eos/eos^		1.19 ± 0.32		3

Significance values: *p < 0.05; **p < 0.01, ****p < 0.0001; n.s. = not significant.

**Table 3.** Quantitative analysis of synaptic density of GlyRα2 in *Glra1*^+/+^ and *Glra1*^spdot/spdot^ neurons

Protein colocalization	Genotype	Synaptic density per 100 microns	Significance and *p*-values	*n* = number of dendrites	*N* = number of experiments
GlyRα2	*Glra1*^+/+^/*Glrb*^eos/eos^	36.13 ± 2.20	*p = 0.0200	113	3
	*Glra1*^spdot/spdot^/*Glrb*^eos/eos^	42.87 ± 2.12		125	3
GlyRα2-gephyrin	*Glra1*^+/+^/*Glrb*^eos/eos^	30.31 ± 2.11	n.s. *p *= 0.7924	106	3
	*Glra1*^spdot/spdot^/*Glrb*^eos/eos^	30.77 ± 1.87		115	3
Extrasynaptic GlyRα2	*Glra1*^+/+^/*Glrb*^eos/eos^	8.42 ± 0.58	****p < 0.0001	101	3
	*Glra1*^spdot/spdot^/*Glrb*^eos/eos^	13.45 ± 0.79		117	3

Significance values: *p < 0.05; ****p < 0.0001; n.s. = not significant

## Results

### GlyRβ protein is widely expressed throughout the brain and colocalizes with GlyRα1

**Figure 1.**
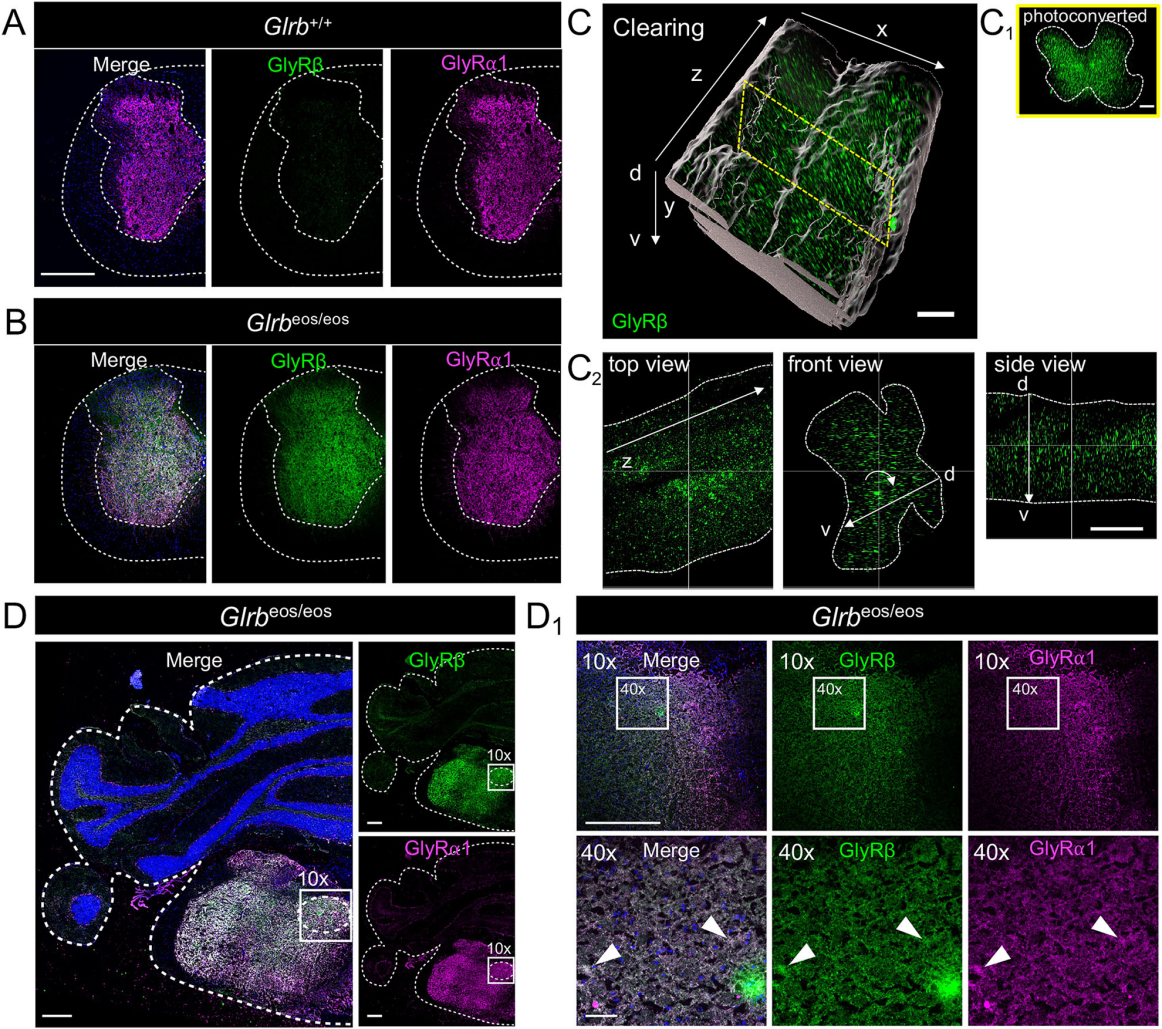
GlyRβ is highly expressed in the spinal cord and brainstem. Immunohistochemical stainings of *Glrb*^+/+^ (***A***) and *Glrb*^eos/eos^ (***B***) spinal cord sections for mEos4b-GlyRβ (green, anti-mEos-488 antibody) and GlyRα1 (magenta, mAb2b). ***C***, mEos4b-GlyRβ (converted mEos4b fluorescence, shown in green) signal in spinal cord tissue of *Glrb*^eos/eos^ after clearing, imaging, and 3D reconstruction (*xyz* represent the axis in 3 dimensions), d, dorsal; v, ventral spinal cord. The gray transparent shell marks the region of interest. Yellow dotted rectangle marks slice of spinal cord shown in ***C***_***1***_. Scale bar refers to 100 µm. (***C***_***1***_) Spinal cord image (front view, for position see yellow dotted rectangle in (***C***) with the photoconverted mEos4b signal (green). Scale bar 100 µm. (***C***_***2***_) Detailed mEos4b-GlyRβ (green) signal with top view (from dorsal), front view (dorsal to ventral axis is marked by a white arrow), and side view (dorsal to ventral axis is marked by a white arrow). Scale bar 200 µm. ***D***, Immunohistochemical stainings of *Glrb*^eos/eos^ brainstem section with higher magnifications (10× and 40×) of the hypoglossal nucleus XII (***D***_***1***_). Stained are mEos4b-GlyRβ (green, anti-mEos-488) and GlyRα1 (magenta, mAb2b). Scale bars refer to 500 µm in the overview images and 10× magnification and 50 µm in 40× magnification.

**Figure 2.**
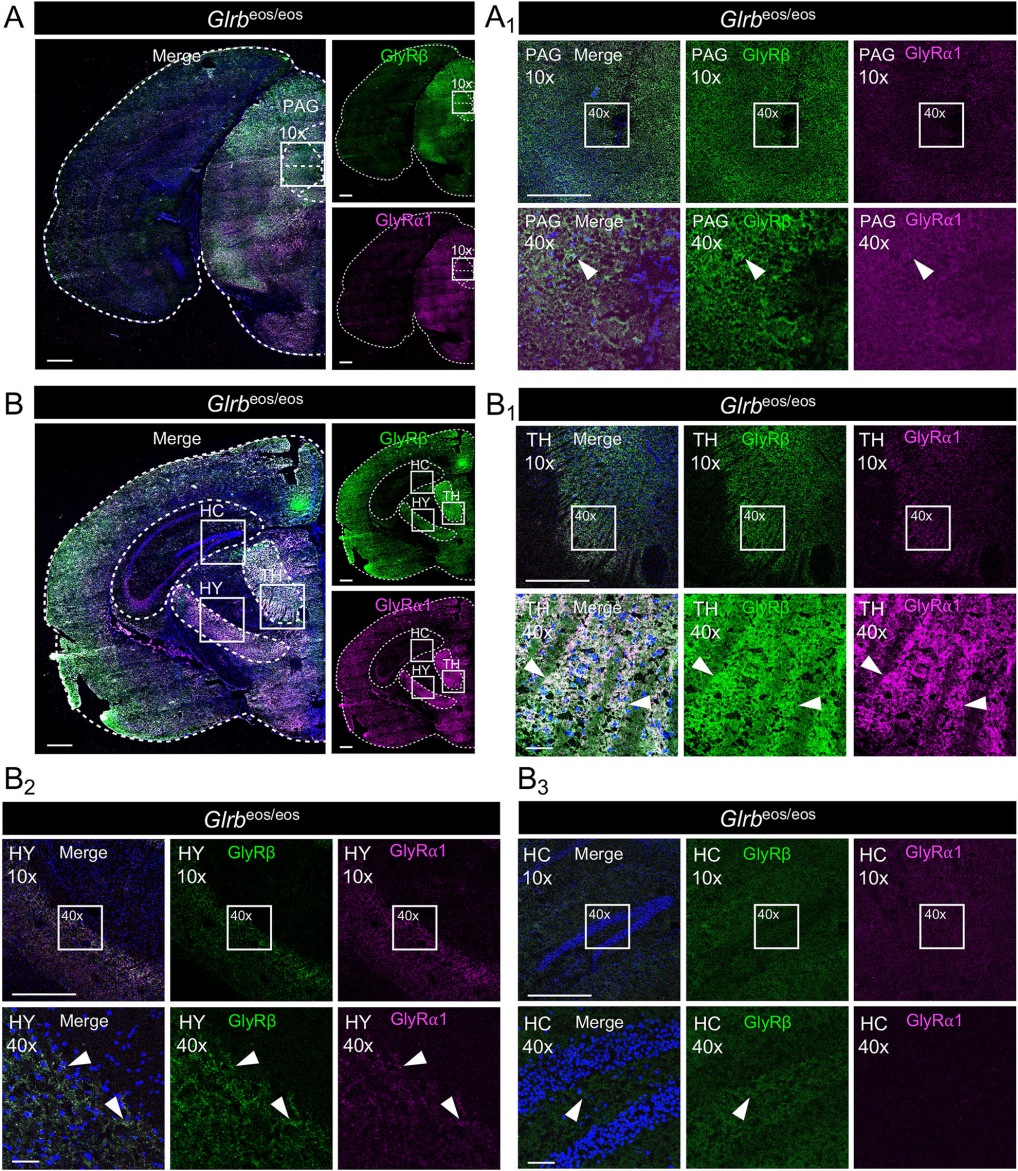
High GlyRβ expression in areas of the periaqueductal gray (PAG) and the thalamic and hypothalamic nuclei. ***A,B***, Immunohistochemical stainings of *Glrb*^eos/eos^ midbrain and forebrain sections with higher magnifications (10× and 40×) of the periaqueductal gray (PAG) and the hippocampus (HC), thalamus (TH), and hypothalamus (HY). Stained are mEos4b-GlyRβ (green, anti-mEos-488) and GlyRα1 (magenta, mAb2b). Scale bars refer to 500 µm in overview and 10× magnification and 50 µm in 40× magnification (***A***_***1***_ PAG; ***B***_***1***_ thalamus; ***B***_***2***_ hypothalamus; ***B***_***3***_ hippocampus).

GlyRβ is responsible for the synaptic localization of the inhibitory GlyR complexes via interaction with the scaffold protein gephyrin (Kneussel and Betz, 2000). While GlyRα1 protein expression is well documented in different BS nuclei, for example, cochlear nucleus, spinal nucleus of the trigeminal nerve, and hypoglossal nucleus (Wenthold et al., 1988), GlyRβ expression has been mainly shown at the mRNA level (Malosio et al., 1991). Recently, a *Glrb*^eos^ knock-in mouse line carrying a GlyRβ allele with an N-terminal mEos4b fluorophore tag (*Glrb*^tm1(Eos4)Ics^, MGI:6331065) was generated allowing the detection of GlyRβ protein expression (Maynard et al., 2021). Following a large-scale screening of the whole mouse brain via vibratome sections of 180 µm thickness, the regions with high GlyR expression were cut in 9-µm-thin cryo-sections and stained by immunohistochemistry, followed by imaging of the GlyRβ and GlyRα1 immunoreactivity (Figs. 1, 2).

As expected, the highest expression levels were observed in SC and BS. GlyRα1 was detected in *Glrb*^+/+^ and *Glrb*^eos/eos^ SC via a commercial GlyRα1-specific antibody (mAb2b). The mEos4b-GlyRβ immunoreactivity was only seen in SC sections of *Glrb*^eos/eos^ mice (Fig. 1*A,B*). Abundant mEos4b signals in the dorsal as well as in the ventral horn of the SC were also observed after the SC clearing, photoconversion of mEos4b with UV illumination, and 3D reconstruction (Fig. 1*C*,*C*_*1*_,*C*_*2*_). GlyRβ was also highly expressed and colocalized with GlyRα1-positive BS nuclei such as the hypoglossal nucleus (Fig. 1*D*, zoomed detail in Fig. *D*_*1*_).

GlyRβ has recently been associated with fear and panic disorders (Deckert et al., 2017; Lueken et al., 2017). Accordingly, we found high GlyRβ protein expression in the midbrain, for example, in the periaqueductal gray (PAG) area, which plays a central role in fear and anxiety circuits (Tovote et al., 2016; Signoret-Genest et al., 2023). The GlyRα1 signals in the PAG were weak, but colocalized with GlyRβ in *Glrb*^eos/eos^ mice (Fig. 2*A*,*A*_*1*_). Additionally, GlyRβ has been detected in parts of the forebrain that were identified as integrating areas and relay nodes in anxiety networks (Kirouac, 2021), for example, the thalamus (TH) and hypothalamus (HY) (Fig. 2*B*,*B*_*1*_,*B*_*2*_). Within the thalamic nuclei, there were differences in GlyRβ expression. The parafascicular nucleus of the intralaminar part of the TH showed strong GlyRβ expression highly colocalized with GlyRα1 (Fig. 2*B*_*1*_). In contrast, GlyRβ was not identified in the lateral geniculate complex and lateral posterior nucleus of the TH. The HY showed a more constant signal for GlyRβ and GlyRα1, as exemplified for the zona incerta (Fig. 2*B*_*2*_).

For the hippocampal formation (HC), there was only a uniform and weak GlyRβ signal, with only few cells stained for GlyRα1 in the CA3 region but not the CA1 region or the dentate gyrus (Fig. 2*B*_*3*_). Therefore, it appears that GlyRβ expression in the hippocampus is relatively low and limited to a few cells.

In summary, a broad protein expression of GlyRβ in many regions of the central nervous system was observed that, in most cases, colocalized with GlyRα1. The strongest signals for both GlyR subunits were found in the SC and BS, which are known to express the highest levels of GlyRs in the CNS (Wenthold et al., 1988).

### GlyRβ expression in gephyrin clusters is unchanged in *oscillator* mice

**Figure 3.**
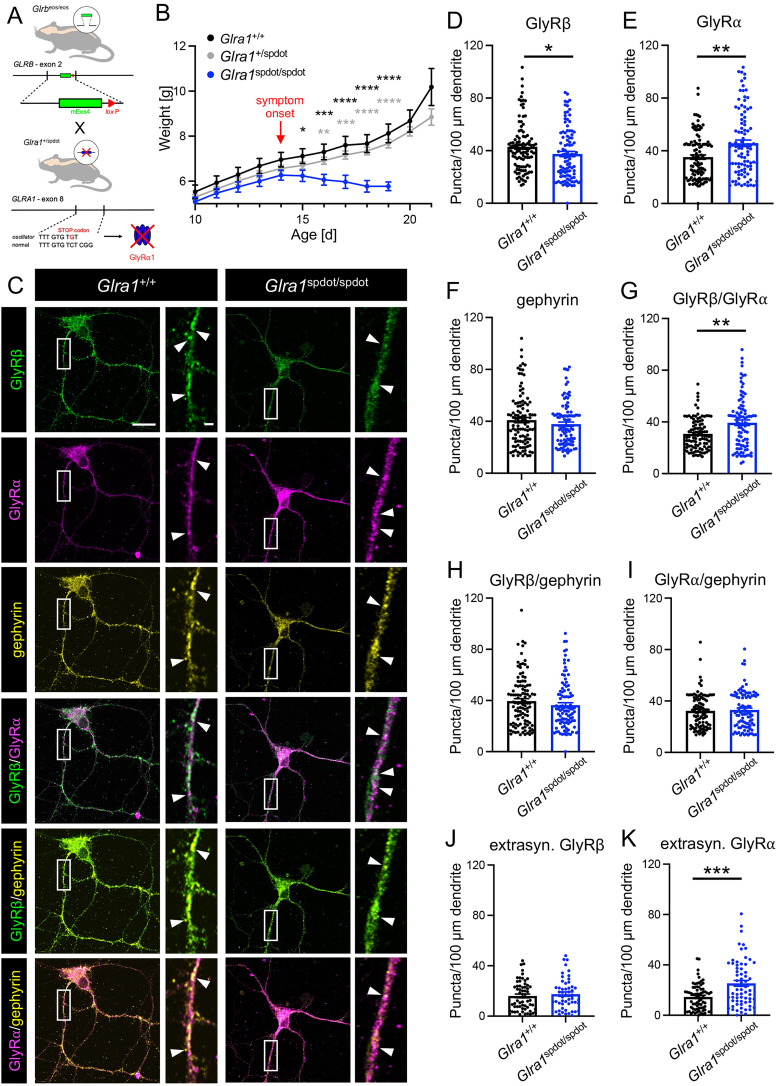
O*s*cillator mice with severe startle disease show unaltered synaptic localization of GlyRβ. ***A***, Breeding scheme of *Glra1*^+/spdot^/*Glrb*^eos/eos^ mice. *Glrb*^eos/eos^ mice have a knock-in of an mEos4b tag in exon 2 of *Glrb* (Maynard et al., 2021). Homozygous *Glra1*^spdot/spdot^ mice harbor a microdeletion in exon 8 of *Glra1* leading to an early STOP codon and thus resulting in a lack of GlyRα1 expression. ***B***, Weight diagram of *Glra1*^+/+^/*Glrb*^eos/eos^ (*n* = 20), *Glra1*^+/spdot^/*Glrb*^eos/eos^ (*n* = 50), and *Glra1*^spdot/spdot^/*Glrb*^eos/eos^ (*n* = 20) mice from day 10 to 21. Marked is the symptom onset of homozygous *oscillator* mice. Note, the number of *Glra1*^spdot/spdot^/*Glrb*^eos/eos^ animals decreased from 20 to 7 after day 14 (onset of symptoms) until day 19 due to premature death. ***C***, Immunocytochemical stainings of mixed primary spinal cord neuronal cultures of *Glra1*^+/+^/*Glrb*^eos/eos^ and *Glra1*^spdot/spdot^/*Glrb*^eos/eos^ mice. Neurons were stained with antibodies against mEos4b-GlyRβ (green), GlyR pan-α (magenta), and gephyrin (yellow). Arrows point to colocalization of stained proteins. Scale bars refer to 20 µm and 2 µm in the magnifications. Quantification of puncta/100 microns dendrite of GlyRβ (***D***), GlyRα (***E***), gephyrin (***F***), GlyRβ–GlyRα (***G***), GlyRβ–gephyrin (***H***), GlyRα–gephyrin (***I***) as well as extrasynaptic GlyRβ (***J***) and GlyRα (***K***) in *Glra1*^+/+^/*Glrb*^eos/eos^ compared to *Glra1*^spdot/spdot^/*Glrb*^eos/eos^ neurons (*N* = 3 independent experiments). Data are shown as mean ± SEM and individual data points. *Glra1*^+/+^/*Glrb*^eos/eos^ *n* = 61–112 and for *Glra1*^spdot/spdot^/*Glrb*^eos/eos^ *n* = 51–102 (*n* = number of dendrites, for detailed information see Table 1). Levels of significance: *p < 0.05, **p < 0.01, ***p < 0.001, and ****p < 0.0001.

The main cause of startle disease in humans are mutations in the *GLRA1* gene. Different spontaneous mutations of *Glra1* in mice have been discovered leading to a similar pathology. To unravel the fate of the GlyRβ subunit and a possible role in the pathophysiology of startle disease, we crossed *Glrb*^eos/eos^ animals (with normal GlyRα1 expression) with *Glra1* mutant mouse strains. A hybrid *oscillator* mouse line was generated that allowed the specific detection of mEos4b-GlyRβ in the absence of GlyRα1 expression (Fig. 3*A*). *Oscillator* represents a *Glra1* null mutation due to a frameshift deletion in exon 8, resulting in an early STOP codon (Buckwalter et al., 1994; Kling et al., 1997). Crossing mice heterozygous for the *oscillator* allele and homozygous for *Glrb*^eos^ resulted in wild-type, heterozygous, and homozygous *oscillator* animals positive for *Glrb*^eos^. Homozygous *oscillator* animals showed severe startle disease. The first symptoms developed around day 14 after birth followed by significant weight loss from day 16 compared to *Glra1*^+/+^ and *Glra1*^+/spdot^ littermates (Fig. 3*B*, at day 16: *Glra1*^+/+^ vs *Glra1*^spdot/spdot^ ***p = 0.0004 and *Glra1*^+/spdot^ vs *Glra1*^spdot/spdot^ **p = 0.0052). Loss of body weight is a typical sign of homozygous animals that display difficulties with suckling and swallowing due to impaired muscle contraction required for these processes. The onset of startle disease symptoms is characterized by tremor, muscle spasms, twitchy tail, stiffness, and poor motor control compared to age-matched littermates. At days 18/19, *Glra1*^spdot/spdot^ mice had to be killed, and the tissues were used for immunohistochemical experiments (Fig. 5). To determine GlyRβ localization, mixed SC cultures (*N* = 3) of *Glra1*^+/+^/*Glrb*^eos/eos^ and *Glra1*^spdot/spdot^/*Glrb*^eos/eos^ neurons were stained for GlyRβ, GlyRα, and gephyrin. A variance analysis for the observed numbers of puncta per 100 µm dendrite for the investigated proteins between different cultures did not reveal any significant changes (*p* = 0.3239; data not shown); hence, the data of the three experiments were pooled without normalization. Since homozygous *oscillator* animals (*Glra1*^spdot/spdot^/*Glrb*^eos/eos^) do not express GlyRα1, a pan-α antibody (mAb4A) was used to stain for all GlyR α-subunits (Fig. 3*C*). The cluster density (puncta per 100 µm dendrite length) was calculated for the individual proteins (GlyRβ, GlyRα, and gephyrin, Fig. 3*D–F*) as well as the colocalization of GlyRβ with GlyRα (Fig. 3*G*) and GlyRβ or GlyRα in gephyrin-positive clusters (Fig. 3*H,I*). While the number of discrete puncta for GlyRβ were significantly reduced in *Glra1*^spdot/spdot^/*Glrb*^eos/eos^ neurons (*p = 0.0275) consistent with previous findings (Maynard et al., 2021), GlyRα labeling showed a significantly increased puncta density in *Glra1*^spdot/spdot^/*Glrb*^eos/eos^ neurons compared to wild type (**p = 0.0060). The overall number of gephyrin puncta was not significantly different between the genotypes (*p* = 0.4663). Interestingly, the colocalization of GlyRβ with gephyrin that represents synaptic but may also include a minor portion of extrasynaptic GlyRβ-positive populations showed no difference between *Glra1*^+/+^*Glrb*^eos/eos^ and *Glra1*^spdot/spdot^/*Glrb*^eos/eos^ neurons (*p* = 0.1976). The same was the case for the colocalization of GlyRα with gephyrin (*p* = 0.78033). The colocalization of GlyRβ and GlyRα in *Glra1*^spdot/spdot^/*Glrb*^eos/eos^, which refers to heteromeric GlyRs, was increased in the same amount as GlyRα alone (**p = 0.0072, Fig. 3*G*, Table 1). Moreover, we looked at extrasynaptic GlyRβ and GlyRα outside of gephyrin signals. Here, we saw no changes in GlyRβ (*p* = 0.5913) but again a significant increase (***p = 0.0009) for GlyRα outside of gephyrin puncta (Fig. 3*J,K*). These findings point to a possible compensation of GlyRα expression in *Glra1*^spdot/spdot^/*Glrb*^eos/eos^ mice. Compensatory mechanisms have been shown previously for the startle disease model *shaky* (Schaefer et al., 2017). Instead, the likely mechanism underlying the enhanced GlyRα expression in *oscillator* mice lacking GlyRα1 could be an increase in GlyRα2 and/or GlyRα3.

**Figure 4.**
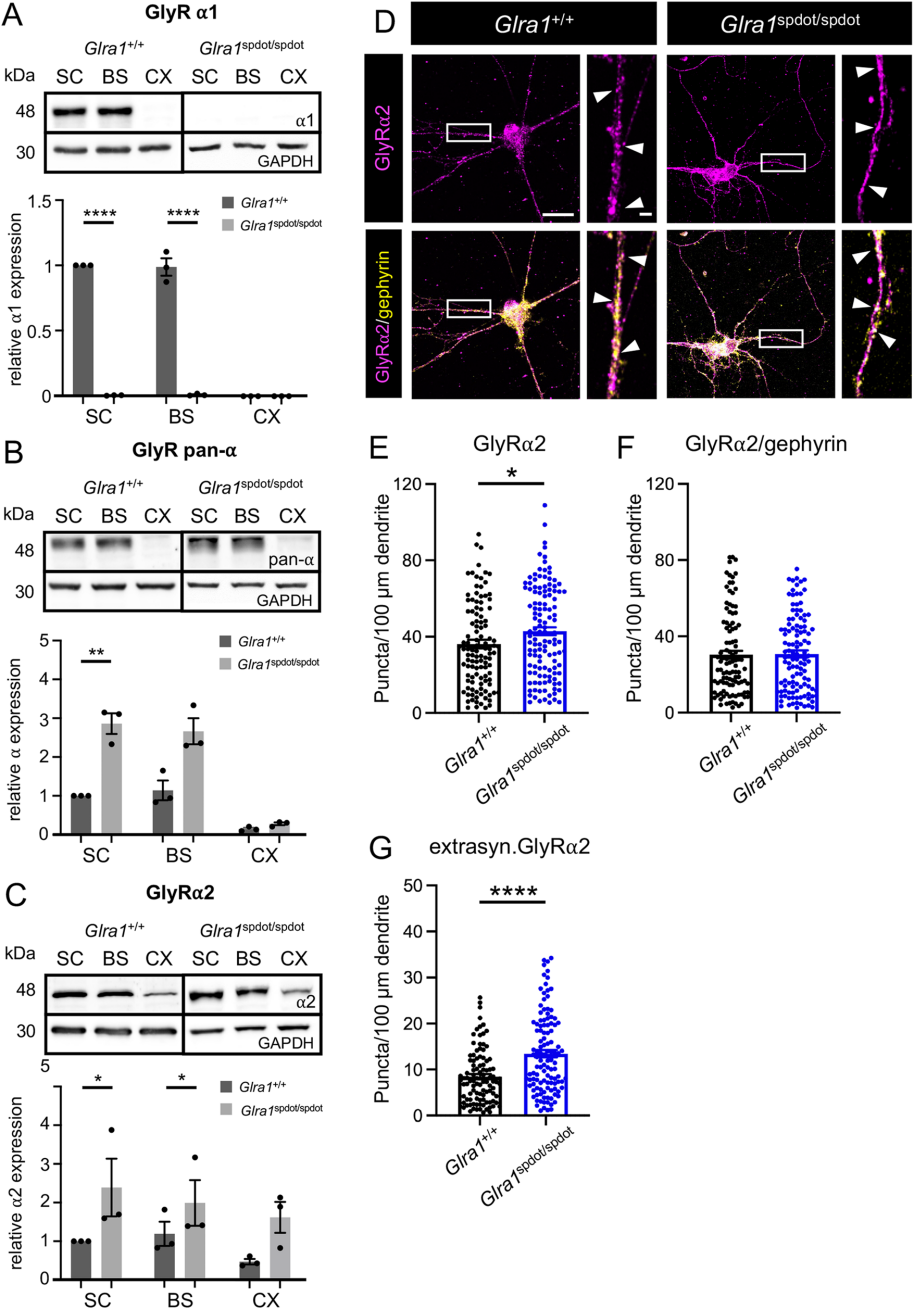
Partial compensation of GlyRα expression by GlyRα2 in *oscillator* mice. ***A–C***, Representative Western blots of spinal cord (SC), brain stem (BS), and cortex (CX) tissue homogenates from *Glra1*^+/+^/*Glrb*^eos/eos^ and *Glra1*^spdot/spdot^/*Glrb*^eos/eos^ mice. Expression of GlyRα1, all GlyRα subunits (pan-α), and GlyRα2 (48 kDa) are shown. GAPDH (30 kDa) served as housekeeping protein. Quantification of normalized expression levels of GlyRα1 (***A***), all GlyRα subunits (pan-α) (***B***) and GlyRα2 (***C***) to GAPDH level in tissue samples of *Glra1*^+/+^/*Glrb*^eos/eos^ and *Glra1*^spdot/spdot^/*Glrb*^eos/eos^ mice. GlyR levels in *Glra1*^spdot/spdot^/*Glrb*^eos/eos^ animals are always shown relative to *Glra1*^+/+^/*Glrb*^eos/eos^ SC samples (*N* = 3). Data are presented as mean ± SEM and individual data points. ***D***, Immunocytochemical stainings of mixed primary spinal cord neuronal cultures of *Glra1*^+/+^/*Glrb*^eos/eos^ and *Glra1*^spdot/spdot^/*Glrb*^eos/eos^ mice. Neurons were stained with GlyRα2 (magenta) and gephyrin (yellow) antibodies. White arrow heads point to colocalization of stained proteins. Scale bars refer to 20 µm and 2 µm in the zoomed images. Quantification of puncta/100 microns dendrite of GlyRα2 (***E***), GlyRα2 in gephyrin-positive clusters (***F***), and extrasynaptic GlyRα2 (***G***). *Glra1*^+/+^/*Glrb*^eos/eos^ *n* = 101–113 and for *Glra1*^spdot/spdot^/*Glrb*^eos/eos^ *n* = 115–125 (*n* = number of dendrites, for detailed information, see Table 3). Level of significance: *p < 0.05, **p < 0.01, and ****p < 0.0001.

To investigate whether GlyRα2 or GlyRα3 expression indeed drives a structural compensation in *oscillator* mice, we analyzed the expression levels of the different GlyRα subunits via Western blots in tissue samples of *Glra1*^+/+^*Glrb*^eos/eos^ (wild-type control) and *Glra1*^spdot/spdot^/*Glrb*^eos/eos^ animals (Fig. 4, Table 2). Our data confirm the loss of GlyRα1 in *Glra1*^spdot/spdot^/*Glrb*^eos/eos^ mice. As shown in Figure 4*A*, GlyRα1 expression is absent in SC, BS, and CX of homozygous *oscillator* mice (SC: ****p < 0.0001, BS: ****p < 0.0001). In wild type, expression levels of GlyRα1 are high in SC and BS and below detection limits in CX, in line with previous mRNA data (Malosio et al., 1991). The overall protein expression of all GlyRα subunits was markedly higher in homozygous *oscillator* animals compared to control (Fig. 4*B*). The pan-GlyRα signals were significantly increased in the SC of *Glra1*^spdot/spdot^/*Glrb*^eos/eos^ mice (**p = 0.0042). A similar trend was observed in BS and CX nearing significance (BS: *p* = 0.0646, CX: *p* = 0.0957) confirming the increase in GlyRα labeling in SC cultures (Fig. 3). Among the GlyRα subunits, a significant increase in GlyRα2 expression in SC and BS of *Glra1*^spdot/spdot^/*Glrb*^eos/eos^ mice (SC: *p = 0.0166, BS: *p = 0.0157) and a strong positive trend in CX (n.s. *p* = 0.0569; Fig. 4*C*) were also detected. The significant increase of GlyRα2 was also confirmed comparing the GlyRα2 puncta along dendrites of SC neurons from *Glra1*^+/+^/*Glrb*^eos/eos^ and *Glra1*^spdot/spdot^/*Glrb*^eos/eos^ mice (*p = 0.02), which reflect most likely extrasynaptic GlyRα2 (****p < 0.0001), while GlyRα2 expression in gephyrin-positive clusters was unaffected (*p* = 0.7924; Fig. 4*D–G*, Table 3). These findings indicate that GlyRα2 (possibly alongside GlyRα3) is partially responsible for compensatory mechanisms in startle disease.

### Persistent GlyRα2 expression in *oscillator* mice does not prevent lethality

**Figure 5.**
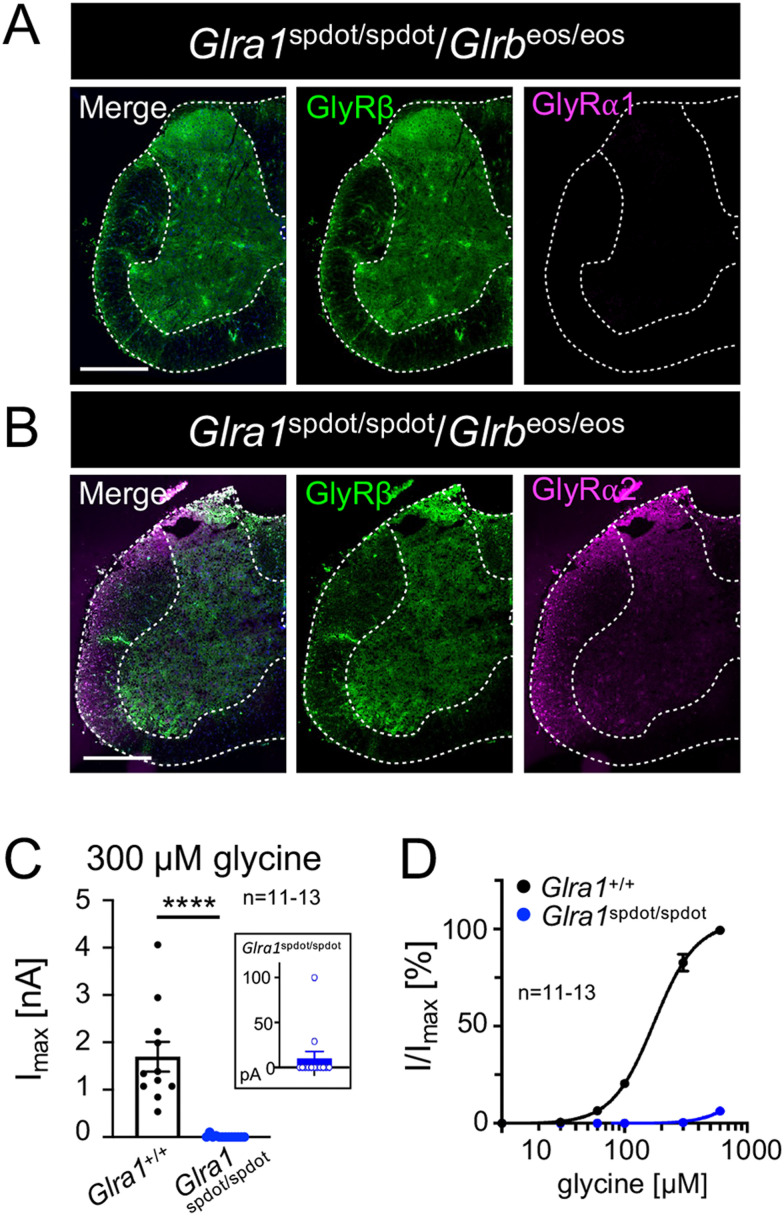
GlyRβ expression is driven by GlyRα2 in *oscillator* mice with severe startle disease. Immunohistochemical staining of slices from *Glra1*^spdot/spdot^/*Glrb*^eos/eos^ mice. Spinal cord (***A,B***) sections stained for mEos4b-GlyRβ (green) and GlyRα1 or GlyRα2 (magenta). Scale bars refer to 500 µm. (***C***) Whole-cell recordings of glycinergic currents from wild-type and *oscillator* spinal cord dissociated neurons at 300 µM glycine. (***D***) Dose–response curves for glycine (3, 10, 30, 60, 100, 300, 600 µM glycine), *N* = 3 independent biological replicates with *n* = 11–13 cells recorded. Level of significance ****p < 0.0001.

Based on the results described above, we conducted a more detailed analysis of GlyR subunit expression. SC cryo-sections of 9 µm thickness were immunolabeled for GlyRβ, GlyRα1, and GlyRα2 (Fig. 5). *Glra1*^spdot/spdot^/*Glrb*^eos/eos^ SC did not express GlyRα1 (Fig. 5*A*), while GlyRα2 was expressed in SC (Fig. 5*B*). Interestingly, although GlyRα2 is expressed and should thus be able to form functional ion channels (Pribilla et al., 1992), whole-cell recordings from neurons of mixed SC *Glra1*^spdot/spdot^/*Glrb*^eos/eos^ cultures revealed almost no function following an application of high glycine concentrations (300 µM, ****p < 0.0001). The lack of functional channels was also seen in dose–response recordings in the *oscillator* mutant compared to neurons from wild-type animals (Fig. 5*C,D*). Although recordings from mixed SC neuronal cultures do not allow to discriminate between neurons from the dorsal or ventral SC, the observed upregulation of GlyRα/α2 (Figs. 3*E*, 4*C–E*) was clearly insufficient to rescue the phenotype and severity of the disease since homozygous *oscillator* animals die at ∼3 weeks of age.

### Localization of GlyRβ in *shaky* mice with a functional *Glra1* missense mutation

**Figure 6.**
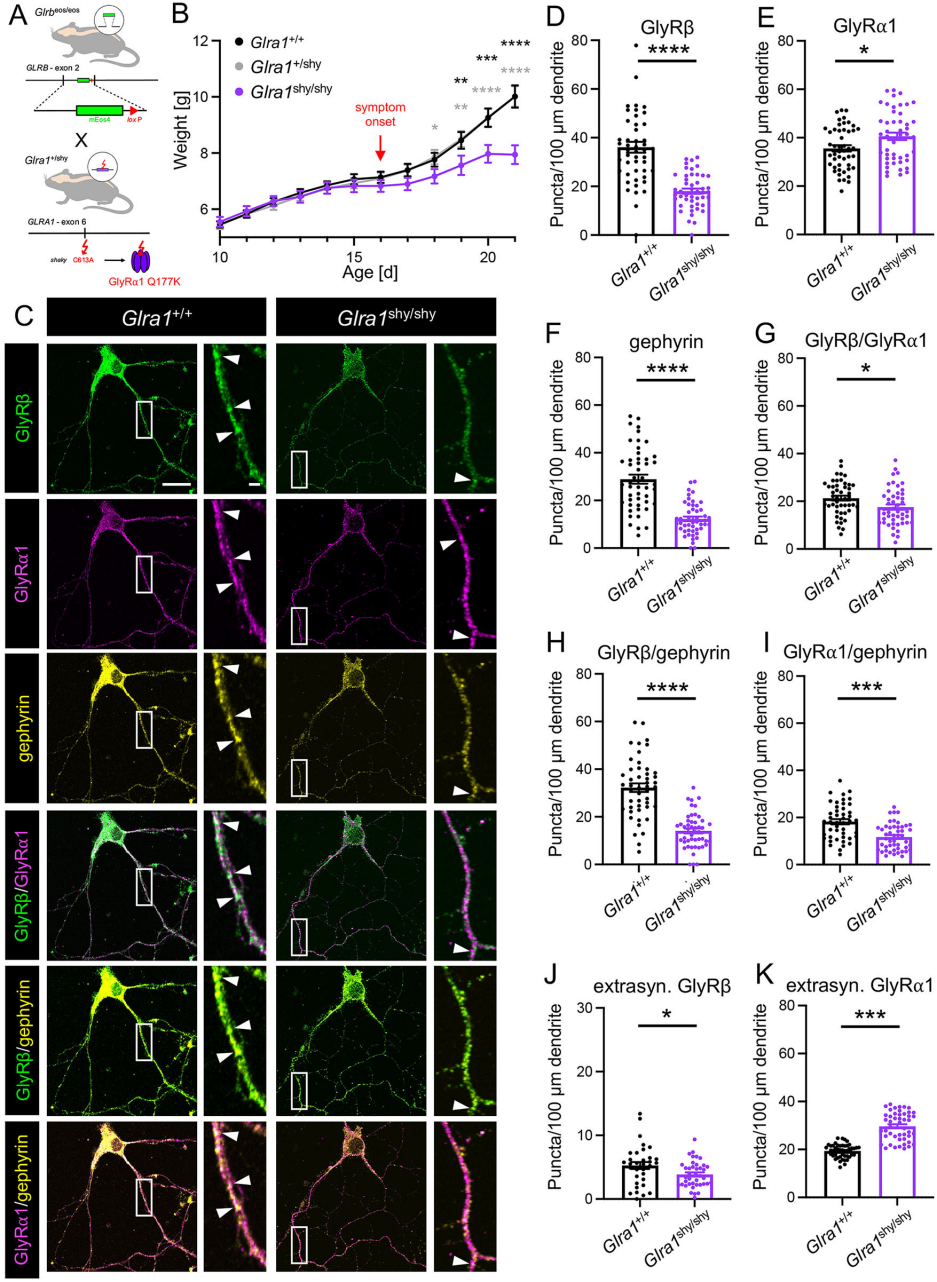
Synaptic localization of GlyRβ in *shaky* mice. ***A***, Breeding scheme of *Glra1*^+/shy^/*Glrb*^eos/eos^ mice. Intercrossings result in animals homozygous for the *shaky* allele harboring a missense mutation Q177K in exon 6 of the *Glra1* gene. ***B***, Weight diagram of *Glra1*^+/+^/*Glrb*^eos/eos^ (*n* = 19), *Glra1*^+/shy^/*Glrb*^eos/eos^ (*n* = 23), and *Glra1*^shy/shy^/*Glrb*^eos/eos^ (*n* = 16) mice from day 10 to 21. Marked is the symptom onset of homozygous *shaky* mice. ***C***, Immunocytochemical stainings of mixed primary spinal cord neuronal cultures of *Glra1*^+/+^/*Glrb*^eos/eos^ and *Glra1*^shy/shy^/*Glrb*^eos/eos^ mice. Neurons were stained with antibodies against mEos4b-GlyRβ (green), GlyRα1 (magenta), and gephyrin (yellow). Colocalization of stained proteins is marked by arrow heads. Scale bars refer to 20 µm and 2 µm in the magnifications. ***D–K***, Quantification of the puncta/100 microns dendrite of GlyRβ (***D***), GlyRα1 (***E***), gephyrin (***F***) alone, GlyRβ–GlyRα1 (***G***), GlyRβ–gephyrin (***H***), and GlyRα1-gephyrin (***I***), as well as the extrasynaptic GlyRβ (***J***), and GlyRα1 (***K***) in *Glra1*^+/+^/*Glrb*^eos/eos^ and *Glra1*^shy/shy^/*Glrb*^eos/eos^ neurons (*N* = 3 independent experiments). Data are shown as mean ± SEM and individual data points (*n* = 33–48 for *Glra1*^+/+^/*Glrb*^eos/eos^ and *n* = 40–48 for *Glra1*^shy/shy^/*Glrb*^eos/eos^, *n* = number of dendrites). Levels of significance: *p < 0.05, **p < 0.01, ***p < 0.001, and ****p < 0.0001.

**Figure 7.**
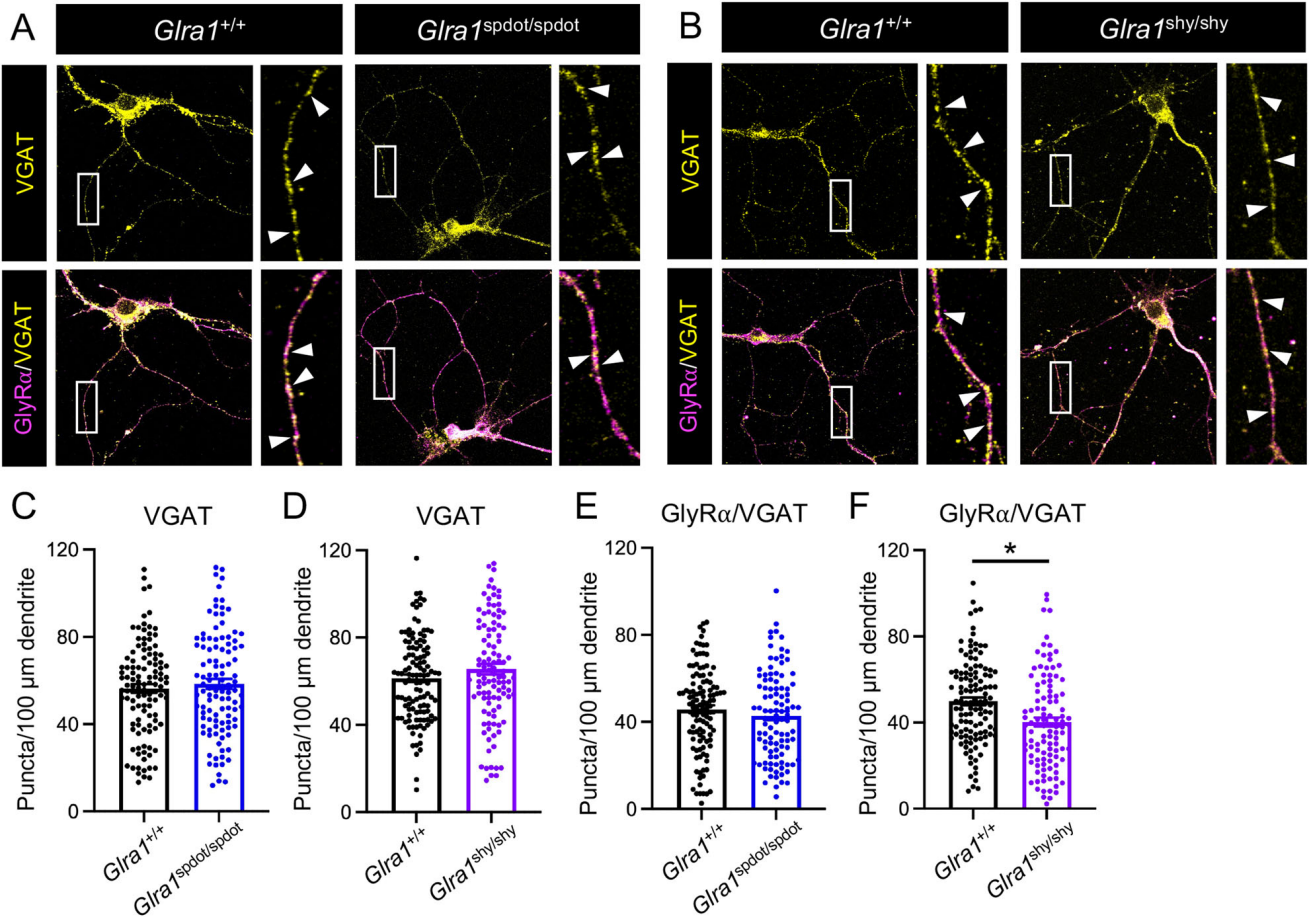
Presynaptic GlyRα in VGAT-positive clusters is only slightly affected in *oscillator* and *shaky* mutant mice. ***A,B***, Immunocytochemical staining of mixed primary spinal cord neuronal cultures of *Glra1*^+/+^/*Glrb*^eos/eos^, *Glra1*^spdot/spdot^/*Glrb*^eos/eos^, and *Glra1*^shy/shy^/*Glrb*^eos/eos^ mice. Neurons were stained with antibodies against GlyRα (magenta) and VGAT (yellow). Colocalization of stained proteins is marked by arrowheads. Scale bars refer to 20 µm and 2 µm in the zoomed images. Quantification of the puncta/100 µm dendrite of VGAT (***C,D***), GlyRα-VGAT (***E,F***) (*N* = 3 independent experiments). Data are shown as mean ± SEM and individual data points (*n* = 113–119 for *Glra1*^+/+^/*Glrb*^eos/eos^, *n* = 101–108 for *Glra1*^spdot/spdot^/*Glrb*^eos/eos^, *n* = number of dendrites). Level of significance: *p < 0.05.

The above experiments suggest that GlyRβ expression as such is unaffected in startle disease caused by the absence of GlyRα1 protein expression. We therefore went on to investigate the GlyRβ expression pattern in another startle disease model, *shaky*. The missense mutation C613A in exon 6 of *Glra1* in the *shaky* mutant leads to a Q177K exchange in the mature protein but does not cause a lack in GlyRα1 protein expression in vivo (Schaefer et al., 2017). To investigate the role of GlyRβ co-assembly with mutant GlyRα1, we crossed the *Glrb*^eos^ mouse line with *Glra1*^shy^ animals (Fig. 6*A*). The presence of the tagged GlyRβ subunit did not change the onset of symptoms on day 16 or the significant weight loss of homozygous *shaky* animals compared to wild-type and heterozygous mice from day 19 on (Fig. 6*B*; day 19: *Glra1*^+/+^ vs *Glra1*^shy/shy^ **p = 0.0093 and *Glra1*^+/shy^ vs *Glra1*^shy/shy^ **p = 0.0042). The number of GlyRβ puncta in mixed SC cultures of *Glra1*^+/+^*Glrb*^eos/eos^ compared to *Glra1*^shy/shy^/*Glrb*^eos/eos^ neurons showed a significant decrease in *Glra1*^shy/shy^/*Glrb*^eos/eos^ neurons for synaptic (****p < 0.0001; Fig. 6*C,D*) as well as extrasynaptic puncta (*p = 0.0263; Fig. 6*J*), while the density of synaptic and extrasynaptic GlyRα1 was significantly increased in *shaky* mice (*p = 0.0341 for synaptic and ****p < 0.0001 for extrasynaptic, respectively; Fig. 6*C*,*E*,*K*). The number of inhibitory synaptic puncta was strongly decreased in *Glra1*^shy/shy^/*Glrb*^eos/eos^ neurons as judged by gephyrin immunoreactivity (****p < 0.0001, Fig. 6*F*), concomitant to the decrease in GlyRβ density. While the number of GlyRβ in GlyRα1-positive clusters was also slightly decreased (*p = 0.0386; Fig. 6*G*, Table 4), GlyRβ and GlyRα1 puncta calculated as the colocalization of the two proteins with gephyrin-positive clusters were significantly reduced (GlyRβ–gephyrin: ****p < 0.0001; GlyRα1-gephyrin: ***p = 0.0002; Fig. 6*H*,*I*). This shows that the higher expression of mutant GlyRα1 in *shaky* mice is not capable of compensating the effects of the mutation while the amount of proper heteromeric GlyR formation at gephyrin-positive sites, most likely mainly synaptic sites, is disturbed. Mutant GlyRα1 may instead form extra- and/or presynaptic homomers less involved in synaptic neurotransmission. Interestingly, colocalization with the presynaptic marker VGAT revealed less total GlyRα in VGAT-positive puncta in SC neurons from *shaky* animals (*p = 0.0494). In contrast, colocalization of GlyRα with VGAT was unchanged between *oscillator* and wild-type SC neurons (*p* = 0.2942; Fig. 7*A–C*, Table 5). Hence, enhanced extrasynaptic GlyRα localization does most likely play a more pronounced role underlying the startle phenotype than increased presynaptic homomers. Taken together, these data suggest that GlyRβ subunits form heteromeric receptor complexes with functionally deficient mutant GlyRα1 subunits in the *shaky* mutant. Trafficking of these complexes to synapses may hinder alternative compensatory mechanisms, for example, an upregulation and integration of functionally-active inhibitory GABA_A_ receptors at synapses.

**Table 4.** Quantitative analysis of synaptic density of *Glra1*^+/+^ and *Glra1*^shy/shy^ neurons

Protein colocalization	Genotype	Synaptic density per 100 microns	Significance and *p*-values	*n* = number of dendrites	*N* = number of experiments
GlyRβ	*Glra1*^+/+^/*Glrb*^eos/eos^	36.08 ± 2.23	****p < 0.0001	48	3
	*Glra1*^shy/shy^/*Glrb*^eos/eos^	18.06 ± 1.07		47	3
GlyRα1	*Glra1*^+/+^/*Glrb*^eos/eos^	35.59 ± 1.28	*p = 0.0341	48	3
	*Glra1*^shy/shy^/*Glrb*^eos/eos^	40.55 ± 1.53		48	3
Gephyrin	*Glra1*^+/+^/*Glrb*^eos/eos^	28.93 ± 1.89	****p < 0.0001	48	3
	*Glra1*^shy/shy^/*Glrb*^eos/eos^	12.25 ± 0.98		48	3
GlyRβ–GlyRα1	*Glra1*^+/+^/*Glrb*^eos/eos^	20.88 ± 1.12	*p = 0.0386	47	3
	*Glra1*^shy/shy^/*Glrb*^eos/eos^	17.54 ± 1.13		48	3
GlyRβ–gephyrin	*Glra1*^+/+^/*Glrb*^eos/eos^	32.22 ± 1.79	****p < 0.0001	48	3
	*Glra1*^shy/shy^/*Glrb*^eos/eos^	14.07 ± 1.06		48	3
GlyRα1–gephyrin	*Glra1*^+/+^/*Glrb*^eos/eos^	17.45 ± 1.18	***p = 0.0002	48	3
	*Glra1*^shy/shy^/*Glrb*^eos/eos^	11.73 ± 0.80		48	3
Extrasynaptic GlyRβ	*Glra1*^+/+^/*Glrb*^eos/eos^	5.26 ± 0.55	*p = 0.0263	33	3
	*Glra1*^shy/shy^/*Glrb*^eos/eos^	3.85 ± 0.32		40	3
Extrasynaptic GlyRα1	*Glra1*^+/+^/*Glrb*^eos/eos^	19.37 ± 0.42	****p < 0.0001	46	3
	*Glra1*^shy/shy^/*Glrb*^eos/eos^	29.65 ± 0.84		48	3

Significance values: *p < 0.05; ***p < 0.001; ****p < 0.0001; n.s. = not significant.

**Table 5.** Quantitative analysis of synaptic density using VGAT in *Glra1*^+/+^, *Glra1*^spdot/spdot^, and *Glra1*^shy/shy^ neurons

Protein colocalization	Genotype	Synaptic density per 100 microns	Significance and *p-*values	*n* = number of dendrites	*N* = number of experiments
VGAT	*Glra1*^+/+^/*Glrb*^eos/eos^	56.37 ± 2.01	n.s. *p* = 0.6831	113	3
	*Glra1*^spdot/spdot^/*Glrb*^eos/eos^	58.46 ± 2.27		108	3
	*Glra1*^+/+^/*Glrb*^eos/eos^	61.31 ± 1.78	n.s. *p* = 0.1595	119	3
	*Glra1*^shy/shy^/*Glrb*^eos/eos^	65.53 ± 2.45		108	3
GlyRα-VGAT	*Glra1*^+/+^/*Glrb*^eos/eos^	45.77 ± 1.85	n.s. *p* = 0.2942	113	3
	*Glra1*^spdot/spdot^/*Glrb*^eos/eos^	42.89 ± 2.02		101	3
	*Glra1*^+/+^/*Glrb*^eos/eos^	41.88 ± 2.02	*p = 0.0494	116	3
	*Glra1*^shy/shy^/*Glrb*^eos/eos^	35.96 ± 2.01		102	3

Significance values: *p < 0.05; n.s. = not significant.

### *Oscillator* but not *shaky* mice exhibit smaller glycinergic synapses

**Figure 8.**
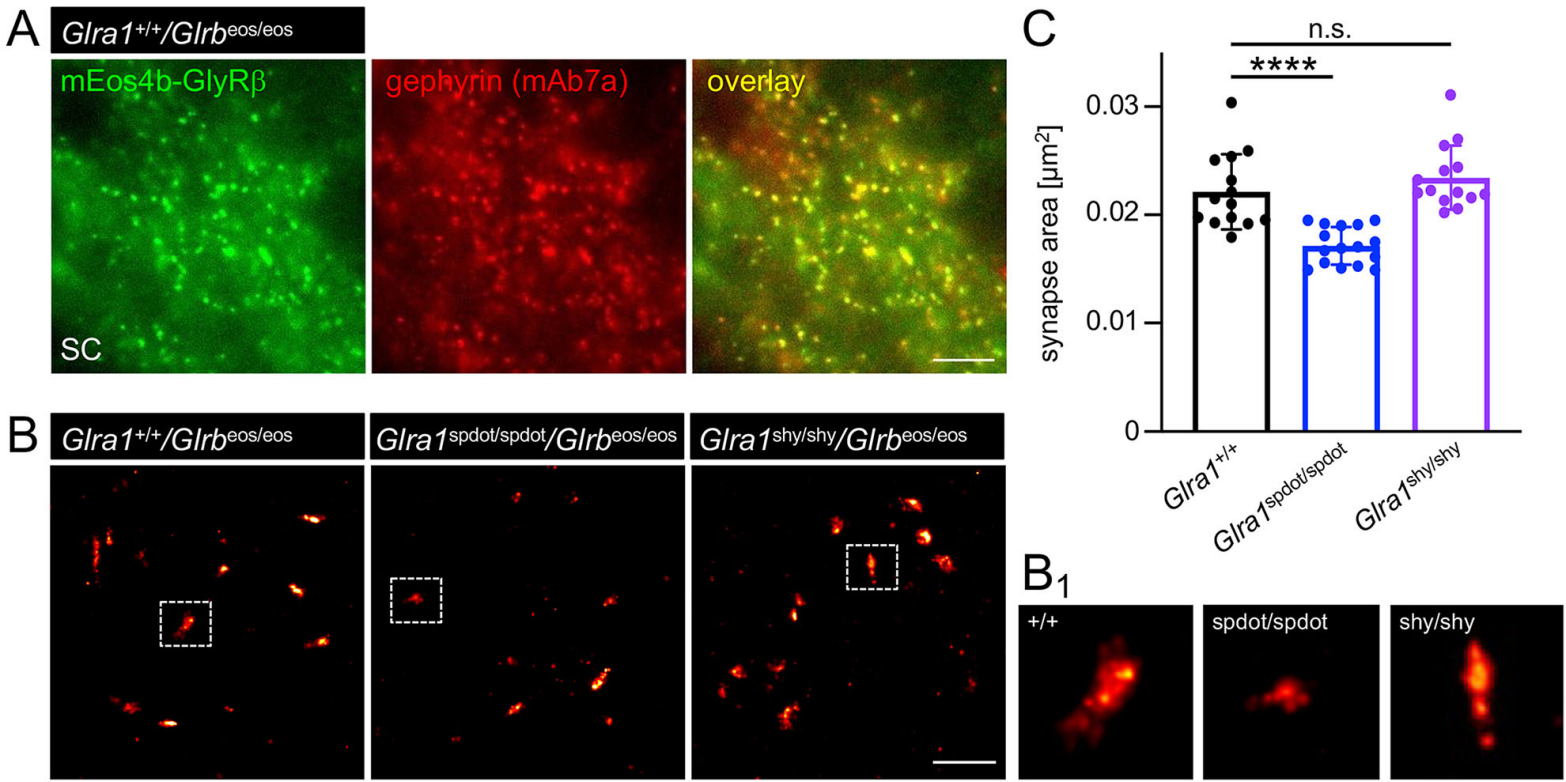
Super-resolution imaging reveals smaller synapse sizes in *oscillator* compared to wild-type and *shaky* mutant mice. ***A***, Spinal cord slice of a wild-type *Glra1^+/+^*/*Glrb*^eos/eos^ mouse (4 µm nominal thickness) expressing fluorescent mEos4b-GlyRβ (green), and immunolabeled for gephyrin (red). Scale bar 5 µm. ***B***, Super-resolution imaging of glycinergic spinal cord synapses in wild-type, *oscillator*, and *shaky* mutants. Representative PALM reconstructions of mEos4b-GlyRβ clusters in spinal cord tissue slices from *Glra1*^+/+^/*Glrb*^eos/eos^, *Glra1*^spdot/spdot^/*Glrb*^eos/eos^ and *Glra1*^shy/shy^/*Glrb*^eos/eos^ mice. Scale bar 1 µm. (B_1_) Enlarged images of dotted squares in ***B***. (***C***) Quantification of mEos4b-GlyRβ cluster sizes in rendered super-resolution images from *Glra1*^+/+^/*Glrb*^eos/eos^ (*n* = 14 fields of view), *Glra1*^spdot/spdot^/*Glrb*^eos/eos^ (*n* = 16), and *Glra1*^shy/shy^/*Glrb*^eos/eos^ mice (*n* = 14) . Significance level ****p < 0.0001; n.s. = not significant.

We carried out super-resolution imaging experiments to further characterize the organization of glycinergic SC synapses in *oscillator* and *shaky* mutant animals. SC tissue was cut into 4-µm-thin slices using a cryostat, immunolabeled, and subjected to PALM. Conventional fluorescence microscopy showed that mEos4b-GlyRβ fluorescence (green) colocalized extensively with gephyrin immunoreactivity (mAb7a, red; Fig. 8*A*). This confirms earlier results according to which the GlyRβ subunit is preferentially targeted to inhibitory SC synapses as a result of its direct binding to gephyrin (Maynard et al., 2021).

For the PALM experiments, we made use of the photophysical properties of the mEos4b fluorophore that is converted from a green to a red fluorescent form upon UV illumination (405 nm). PALM super-resolution images were reconstructed by rendering the single molecule detections with a Gaussian intensity profile corresponding with the localization precision of the detections (Fig. 8*B*). The rendered images were then analyzed using the spot detector plugin in Icy to calculate the 2D area of the synapses. Given the stereotypic clustering of GlyRs at inhibitory SC synapses (Maynard et al., 2021), there is a close correspondence between the synapse size and receptor numbers. In other words, the synapse size is a useful readout for the quantification of GlyR clustering at glycinergic synapses.

According to our results, the size of glycinergic synapses was significantly reduced in *Glra1*^spdot/spdot^ animals compared to wild-type controls (****p < 0.0001, Fig. 8*C*), suggesting that the lack in full-length GlyRα1 subunits and the concomitant reduction in heteromeric GlyR complexes causes a shrinkage of the synapse with likely functional consequences (Fig. 8*B*,*B*_*1*_). In contrast, the synapse sizes in the *shaky* mutant were indistinguishable from wild-type littermates (*p* = 0.3693, Fig. 8*C*). This indicates that the full-length GlyRα1 *shaky* mutant assembles with mEos4b-GlyRβ to form heteropentameric receptor complexes that are targeted to functionally deficient synapses (Schaefer et al., 2017). The *oscillator* and *shaky* mutations thus lead to very different structural and functional consequences, respectively.

## Discussion

In this paper, the *Glrb*^eos^ mouse line (Maynard et al., 2021) was used to investigate the role of GlyRβ in severe startle disease caused by GlyRα1 mutations. *GLRA1* encoding GlyRα1 is the most commonly affected gene underlying startle disease in humans. Dominant mutant GlyRs are generally expressed at normal levels, but show altered receptor function. In contrast, recessive mutants often display disturbed protein biogenesis (Bode and Lynch, 2014; Schaefer et al., 2022) pointing to alternative pathophysiological mechanisms. Recent in vitro studies included the GlyRβ subunit in functional assays to better understand a possible contribution of this subunit to inhibitory signal transduction processes. These studies identified increased maximal currents, accelerating decay rates but also slower decay of glycinergic currents (Chung et al., 2010; Zhang et al., 2015; Wang et al., 2018). Functional recordings in neurons from mouse models of startle disease with unaffected GlyRβ but a mutation in GlyRα1 showed reduced mIPSC amplitudes and frequencies but opposite effects on decay times of mIPSCs depending on the mouse model used (*oscillator* or *spasmodic*) (Graham et al., 2006).

The role of non-mutated GlyRβ in the presence of GlyRα1 startle disease variants for receptor localization at synapses in vivo is only partially understood. GlyRα1 and GlyRβ form the adult heteromeric synaptic receptor complex together with gephyrin as scaffold protein (Betz, 1991; Specht et al., 2011; Xiong et al., 2014; Patrizio et al., 2017). Investigations of the GlyRβ subunit at the protein level were mainly indirect, using gephyrin labeling to mark postsynaptic sites. The *Glrb*^eos^ mouse model makes it possible to label GlyRβ at protein level directly and investigate GlyRβ localization in the whole mouse brain and SC. In agreement with earlier reports (Weltzien et al., 2012; Maynard et al., 2021), we detected the highest expression level of GlyRβ in SC where it is widely distributed throughout the dorsal and ventral horn, and in the BS, especially in the hypoglossal nucleus (cranial nerve XII). We have also observed GlyRβ expression in higher brain areas, for example, even in areas with low levels or lack of GlyRα1, the major synaptic partner of GlyRβ in the adult central nervous system. GlyRβ is highly expressed in the forebrain in line with the reported expression of the *Glrb* transcript in this area (Fujita et al., 1991; Malosio et al., 1991), for example, in the thalamus and more specifically in the parafascicular nucleus of the intralaminar part and in the hypothalamus. The thalamus and hypothalamus were also positive for GlyRα1, arguing for synaptic heteromeric GlyR complexes in these brain areas. The presence of GlyRs in the thalamus and hypothalamus by immunohistological stainings using antibodies against GlyRα1/α2 has been shown previously (Rampon et al., 1996). Moreover, a specific functional contribution of GlyRs to fast inhibitory neurotransmission was identified in the thalamic brain slice recordings in the presence of strychnine (Ghavanini et al., 2005). In the midbrain, we found GlyRβ protein expression in the PAG that plays a central role in fear and anxiety circuits (Tovote et al., 2016; Signoret-Genest et al., 2023). This is in line with a recently reported association of GlyRβ with fear and panic disorders (Deckert et al., 2017; Lueken et al., 2017). In contrast to GlyRβ, GlyRα1 was only sparsely found in the PAG, arguing that the glycinergic contribution in fear and anxiety circuits occurs predominantly through other GlyRα subunits such as GlyRα2 serving as partner for GlyRβ in the PAG. Changes in GlyRβ expression level have been proposed to underlie an association of GlyRβ with fear. However no anxiety-like behavior has been reported in *spastic* mice harboring <10% of full-length GlyRβ (Kingsmore et al., 1994). In contrast, altered anxiety-related behavior characterized by massively increased startle reactivity was identified in *spasmodic* mice that carry a *Glra1* missense mutation A52S leading to functional changes in glycine affinity but no alteration in GlyRβ expression (Schaefer et al., 2020). Hence, changes in the expression level alone are unable to explain changes in behavioral patterns under disease conditions. At the synaptic level, adaptations and modulation of structural receptor organization, receptor mobility, and changes in localization between synaptic and extrasynaptic sites (Specht et al., 2013) may provide mechanistic insights into the regulatory role of GlyRβ, including ways to trigger compensatory mechanisms in startle disease.

To investigate the impact of GlyRβ in startle disease due to distinct *Glra1* mutations, *Glrb*^eos^ animals were crossed with heterozygous *oscillator* and *shaky* mice. *Glrb*^eos^ had no impact on the lethality of the homozygous carrier of the *oscillator* and *shaky* alleles which died at the reported time points [*oscillator* 3 weeks; *shaky* 4–6 weeks (Buckwalter et al., 1994; Schaefer et al., 2017; Schaefer et al., 2018)]. *Oscillator* mice lack GlyRα1 while *shaky* animals express mutated GlyRα1 with impaired function. A lower GlyRβ clustering in *oscillator* animals was therefore expected. However, we also identified a significant increase of the total GlyRα level, most likely driven by the GlyRα2 subunit. Both α2 and α3 subunits are expressed in the SC (Harvey et al., 2004). The formation of GlyRα2β protein assemblies with gephyrin in the ER, trafficking along microtubules and membrane insertion points and clustering at postsynaptic sites (Maas et al., 2006; Kneussel and Loebrich, 2007), appears to be unaffected in *oscillator* mice. GlyRα2 has its highest expression at embryonic stages in rodents and is downregulated after birth in the SC (Liu and Wong-Riley, 2013), while in other forebrain areas, GlyRα2 persists as the dominant α subunit (Jonsson et al., 2012). The developmental switch from GlyRα2 to GlyRα1 expression in the SC in vivo has been suggested to accompany the onset of startle disease symptoms (Schaefer et al., 2022). The observed upregulation of GlyRα2 may therefore constitute a compensatory attempt by localizing GlyRα2β at synaptic sites similar to the persistance of GlyRα2β complexes in areas of the forebrain. Although functionality of GlyRα2β has been demonstrated in transfected HEK-293 cells (Pribilla et al., 1992) and forebrain (Kilb et al., 2008), the whole-cell recordings from hypoglossal neurons of homozygous *oscillator* mice displayed only residual glycinergic currents with dramatically reduced amplitudes and frequencies (Graham et al., 2006). In line with these data, whole-cell recordings from SC motoneurons of homozygous *oscillator* mice in this study also revealed only small residual glycinergic currents.

The reduced size of synapses observed by SMLM in homozygous *oscillator* animals is a likely consequence of the lack of GlyRβ containing receptors that are necessary for the formation and maintenance of the synaptic structure. Interestingly, smaller synapses specifically in the ventral horn have also been characterized in heterozygous *oscillator* animals (Maynard et al., 2021), suggesting that a reduction by ∼50% of GlyRα1 has a similar deleterious effect for glycinergic synapse structural integrity in vivo. According to our results, the upregulated GlyRα2 might preferentially form homomeric receptors or, together with GlyRβ, heteromeric extrasynaptic receptors unable to compensate for the lack of the fast synaptic kinetics of GlyRα1 at SC synapses, and thus inevitably result in lethality. The slow activation kinetics (Mangin et al., 2003) and longer durations (Krashia et al., 2011) than other GlyR subtypes are also not consistent with a synaptic function of GlyRα2.

In the *shaky* mutant, GlyRα1^Q177K^ is upregulated in homozygous animals. This argues that the mutation does not severely affect folding of the receptor, as unfolded or incorrectly-folded protein assemblies are usually subjected to ER-associated proteasomal degradation (Valkova et al., 2011). Our data strongly suggest that the mutant GlyR complexes are instead transported together with GlyRβ and gephyrin to synaptic sites. Synaptic integration is supported by cluster density analysis showing less GlyRβ and gephyrin clusters in general and consequently lower localization of both GlyRβ and mutated GlyRα1 in gephyrin-positive clusters in cultured neurons. Moreover, the upregulated GlyRα1 might also result in larger numbers of homomeric and functionally impaired GlyRα1 complexes at extrasynaptic sites. Despite the reduced total number of GlyRα1β clusters in culture, however, we did not see any obvious changes in synapse size in native SC tissue from homozygous *shaky* mice.

In addition, the upregulation of mutant GlyRα1 may also impact presynaptically localized GlyRs, which result in slightly depolarizing glycine currents regulating the release of glycine from presynaptic terminals (Xiong et al., 2014). A consequence of severely impaired homomeric presynaptic GlyRα1 in *shaky* animals would be a lower glycine release from presynapses which impacts the startle disease pathology in this mutant.

The role of unaffected GlyRβ can be interpreted in two directions. On the one hand, the presence of mutant GlyRβ-containing receptor complexes at synapses could be detrimental in that it prevents the compensation by other GlyRα subunits through the integration of functional GlyRs. On the other hand, mutant receptors could also play a beneficial role, since they may help to preserve the structural integrity of the synapses, notably the postsynaptic gephyrin scaffold. Consequently, gephyrin clusters would be kept in place that could then trap other receptors or adaptor proteins. In contrast, the loss of inhibitory synapses in the *oscillator* mutant makes functional compensation less likely. Nonetheless, in the models of severe startle disease studied here, neither mechanism can efficiently overcome lethality.

## Abbreviations

GlyRs, glycine receptors; GlyT2, glycine transporter 2; ECDs, extracellular domains; TM, transmembrane; *Glra1^+/+^*, wild-type mice; *Glra1*^spdot/spdot^, *oscillator* mice; *Glra1*^shy/shy^, *shaky* mice.
